# How perceptions of a successful physician-scientist varies with gender and academic rank: toward defining physician-scientist's success

**DOI:** 10.1186/s12909-020-1960-9

**Published:** 2020-02-13

**Authors:** Ruth Gotian, Olaf S. Andersen

**Affiliations:** 1 Mentoring Academy, Weill Cornell Medicine, 525 East 68th Street, Box 124, New York, NY 10065 USA; 2 Department of Anesthesiology, Weill Cornell Medicine, 525 East 68th Street, Box 124, New York, NY 10065 USA; 3 Weill Cornell/Rockefeller/Sloan Kettering Tri-Institutional MD-PhD Program, New York, NY USA; 4 Physiology and Biophysics, Weill Cornell Medicine, New York, NY USA

**Keywords:** Physician-scientist, Success, MD-PhD, Gender, Faculty, Promotion

## Abstract

**Background:**

Physician-scientists (the physician-scientist workforce) are aging, and there are too few physician-scientists in the pipeline to replace those who retire. Moreover, the pipeline is leaky because some trainees and junior physician-scientists choose other career paths. Significant attention has been directed toward patching the leaking pipeline, thereby increasing the quantity of physician-scientists. Less attention has been devoted to identifying and training more successful physician-scientists, thereby increasing the quality of the pool and making up for the attrition. Though all training programs strive to develop more successful graduates, there is no clear understanding of what constitutes predictors of future success. Identifying characteristics of success would enable those who recruit trainees—and later hire and fund physician-scientists—to make more informed decisions. It also could impact on the training, as it would be possible to focus on competencies that foster success. Predictors of success are therefore important. Prior to taking on this task, however, we must first define success for physician-scientists.

**Methods:**

To identify likely characteristics of success, we undertook a qualitative case study where 21 physician-scientists were interviewed to determine their perceptions of what constitutes a successful physician-scientist. Sixteen interviewees were selected based on convenience sampling, while the remaining five were selected based on the snowball effect. Interviews were transcribed and coded in Dedoose® and a qualitative analysis was conducted using an inductive approach to content analysis.

**Results:**

There was considerable variation in their perceptions based on seniority and gender. Junior physician-scientists focused on metrics on which their promotion is based, e.g., publications and grants; senior physician-scientists focused on their legacy, e.g., contribution to the field and mentoring. Women were more likely to emphasize objective measures of success, like publications, while concurrently concentrating on relational skills, like networking, collaboration and public recognition. Men emphasized the impact of science and subjective characteristics like boldness, confidence and critical thinking.

**Conclusion:**

Clearly, physician-scientists are not working off of a uniform metric of success, thereby making their evaluation and remuneration a convoluted process, especially if those evaluating the physician-scientists are not of the same mind as to the definition of success.

## Background

**Fig. 1:**
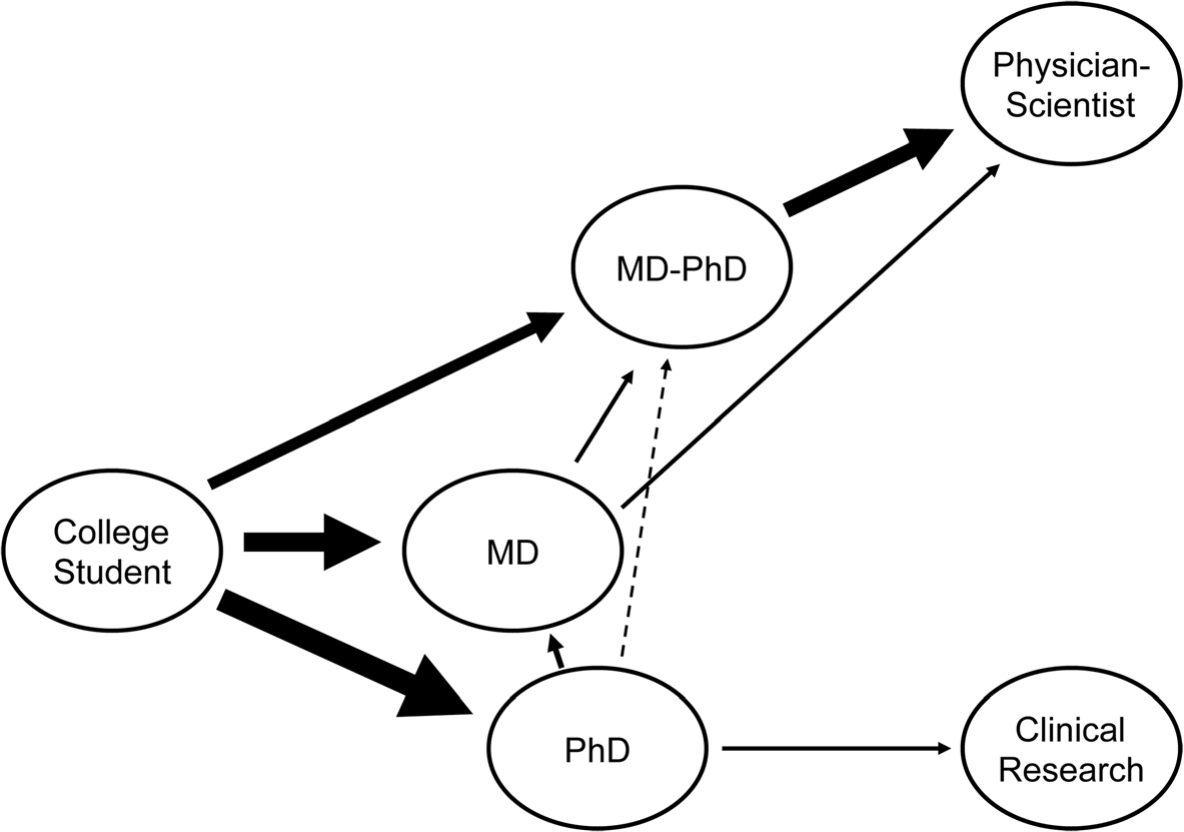
Pathways to become a physician-scientist. The figure depicts the pathways open to college students in the US; it does not include time a student may spend on post-baccalaureate activities, such as working as a technician in a research laboratory. The most straightforward path is to matriculate into an MD-PhD Program and pursue postgraduate clinical and research training after graduation from the MD-PhD Program. Some students may not be aware of the combined-degree path, or may not be ready to commit to the length training (8 years on average), and matriculate into MD programs, where they become interested in pursuing a research career; they may then be able to transfer into an MD-PhD program affiliated with their medical school, or they may graduate and pursue more extensive postdoctoral research training. Other students want to pursue a research career, but become interested in translational/clinical research during/after their PhD training. They can matriculate into MD programs and pursue postgraduate clinical and research training after receiving their MD. Transfers from PhD to MD-PhD programs tend to be rare, as reflected in the stippled path

There is a continued need to train creative and committed physician-scientists, defined here as individuals who have an MD and the research training required to be successful scientists. This is critical in order to maintain the momentum in research advances and to catalyze the transfer of scientific knowledge and discovery between laboratory and patients, thereby improving the nation’s health. There are numerous training paths to prepare individuals for a physician-scientist career (Fig. 1), including the combined MD-PhD Program and MD training with focused research training (with or without the PhD) [1]. The focus of our study is on physician-scientists trained in the United States of America (US), where MD and MD-PhD students begin their training after 4 years of undergraduate education. The combined-degree students receive their training for the two degrees concurrently in programs that are charged with developing an integrated learning environment. The trainees thus are prepared for careers as physicians, who have active research programs. To our knowledge, this model differs from that in most other countries. In the US, the majority of physician-scientists come through the National Institutes of Health-funded Medical Scientist Training Program (MSTP) and hold the dual MD and PhD degrees [1–3].

Successful physician-scientists are both problem posers and problem solvers. As physicians, they are skilled in caring for patients, which enables them to identify important unmet needs and identify patients with a constellation of symptoms and findings that “do not make sense,” the patients that stimulate new research. As scientists, they have acquired the expertise and biological insights that enable them to translate “do not make sense” into plans for new research to explore the underlying mechanism(s). Taken together this combination of skills makes physician-scientists particular important contributors to the laboratory and clinical advances that eventually lead to improvements in the nation’s health.

Predicting future success of physician-scientists, as trainees and later as investigators, is an ongoing challenge for those who train, hire and fund them. Furthermore, we are not aware of a consensus definition of success. Considerable effort is invested in tracking the future careers of physician-scientists, at least the subgroup that graduated from MD-PhD programs, but deciding what constitutes a successful career remains elusive—and is likely to change with time. This is due to the changes in a physician-scientists’ career and the changing needs of society over time. How to structure the training of physician-scientists and predicting their success thus becomes a moving target [4–7]. To focus this study, we decided to concentrate on graduates of the NIH MSTPs. Because nearly 80% of US MD-PhD graduates are alumni of MSTPs, we felt this was a representative sampling that does not limit the generality of the study [2]. Focusing on the dual-degree MD-PhD subpopulation of physician-scientists, the consensus seems to be that the programs are successful, even if no one has devised a definition of success.

We therefore need to develop an understanding of what is perceived to constitute success. As a first step in this endeavor, we decided to ask the following questions which served as both the research and interview questions:

1. What is considered an exemplar/successful physician-scientist?
2. What factors contribute to the success of a physician-scientist?

We approached these questions in a national study where 21 physician-scientists, all with dual MD-PhD degrees, were interviewed to determine what they considered to be success (as a physician-scientist).

As we were aiming to identify the perception of success among physician-scientists, we explored this question using the qualitative research method of interviews, which allowed us to identify and explore the underlying meaning related to a particular perspective, experience or culture of individuals or groups [8–16].

## Methods

To obtain a valid, uniform, representation of participants via purposeful sampling, a hallmark of qualitative research [17], we used the following criteria:

1. All participants were physician-scientists with combined MD and PhD degrees;
2. All participants were trained in a US-based, NIH-funded Medical Scientist Training Program.
3. All participants had completed their MD-PhD training, as well as their residency and/or fellowship training, and had faculty appointments.
4. To the extent possible, the demographic sampling of the interviewed pool mirrored the national demographic of race and gender for physician-scientists.

During a three-month period in the fall of 2015, 25 physician-scientists who met the aforementioned criteria were asked to participate in the study. Of this group, 21 physician-scientists at various stages of their career agreed to be interviewed by one of us (RG) for 30–60 min, either by phone or face-to face, in order to determine their perception of what is a successful physician-scientist. An initial group of 16 physician-scientists was selected by one of us (RG) based on her knowledge of this cohort founded on her two decades of experience with physician-scientists and scrutinized by the other (OSA), a physician-scientist and former Chair of the National MD-PhD Association. The 16 physician-scientists were chosen based on the aforementioned criteria, with an eye toward having a demographic which mirrored the national pool of physician-scientists [2]. The remaining five participants were identified based on the recommendations of other physician-scientists, a method commonly referred to as the “snowball effect” [8, 18]. Data saturation was achieved after 13 interviews, but the study was continued to ensure representative sampling.

Among the 21 physician-scientists who participated in the study there were 16 (76%) men and five (24%) women. One (5%) was Asian, two (10%) were African-American and the rest (85%) were Caucasian. Their graduation years ranged between 1969 and 2008. Their current employment institutions were predominantly private or Ivy-League academic medical centers. One participant worked at an undergraduate Historically Black College/University. Eight (38%) were Junior Faculty (one (5%) Instructor, seven (33%) Assistant Professors); five (24%) were Associate Professors and eight (38%) were Professors. The latter group includes individuals with significant administrative responsibilities, e.g., Department Chair, Division Chief, or Dean in addition to their research and clinical responsibilities. (Two Associate Professors and one Assistant Professor also had administrative obligations). The four physician-scientists who ultimately did not participate in the study were all Caucasian; three were male and one was female.

The participants were asked two questions:

1. What is considered an exemplar/successful physician-scientist?
2. What factors contribute to the success of a physician-scientist?

The interviews were transcribed and coded in Dedoose® by one of us (RG). As there is no current definition of success to test this against, we used an inductive approach to content analysis [19]. Using a theme frequency distribution chart, the responses were chronicled and analyzed based on themes that described characteristics of success. Any measure of success that was identified by the participants was recorded. Characteristics with only one mention were not included in the analysis. The same method was used for all interviews. Based on this inductive approach, we were able to move the data from specific responses to general categories, and then combine them into a larger whole and general statement.

To guard against undue bias and ensure rigor, two experts in adult learning, with no known association with physician-scientists training (so-called “sophisticated barbarians” [20]), also coded the data and assessed our data analysis. The data were analyzed separately, and they each discussed their impressions with RG. These discussions were important for identifying gender and career stages as important variables when discussing perceptions of success as all three coders noticed the same themes.

## Results

**Fig. 2:**
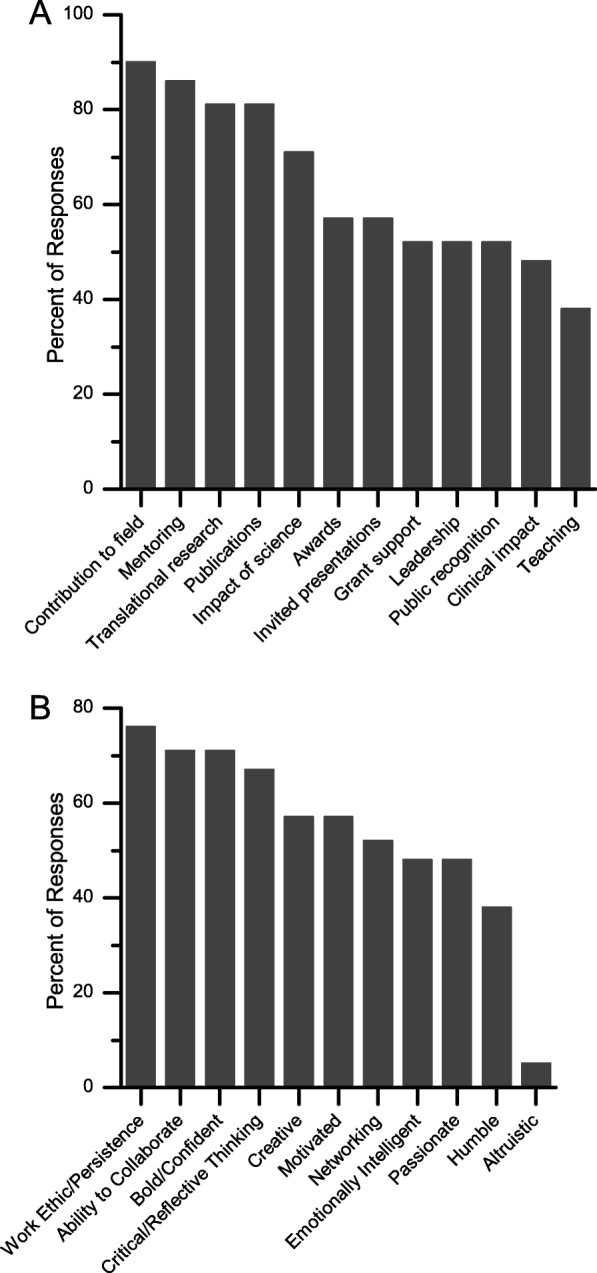
Rank order of the frequency that measures of success were mentioned by all study participants **a** objective measures. **b** subjective measures

The reported measures of success fell into two broad categories of subjective and objective measures. Additional file 1: Tables S1 and S2 list all subjective and objective measures of success that could be identified. Figure 2a summarizes the information on the objective descriptors; Fig. 2b summarizes information on the subjective descriptors.

Combining the information in Fig. 2a and b, we find that there is higher consensus among the objective than the subjective measures of success. More than 75% of the participants identified four objective and one subjective measures of success: Contribution to field; mentoring; engage in translational research; quantity and quality of publications; strong work ethic/persistence.

### Effect of gender and academic rank

**Fig. 3:**
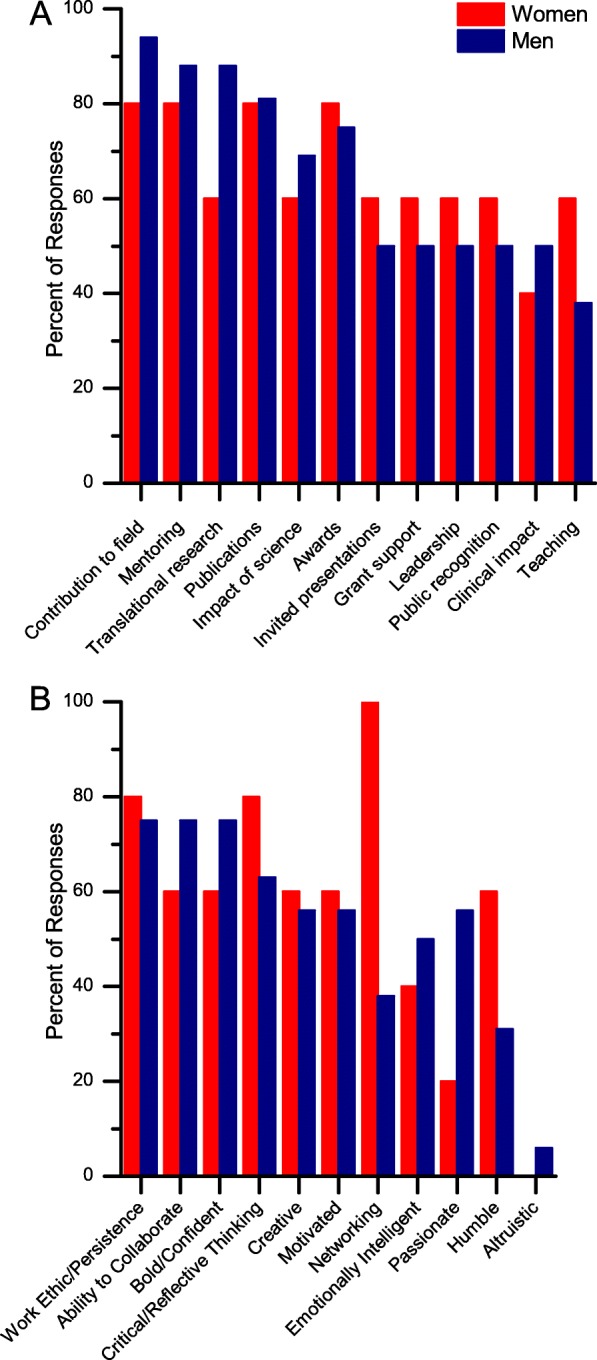
Comparison of the participants' perception of **a** objective measures and **b** subjective measures of success varied based on gender

**Fig. 4:**
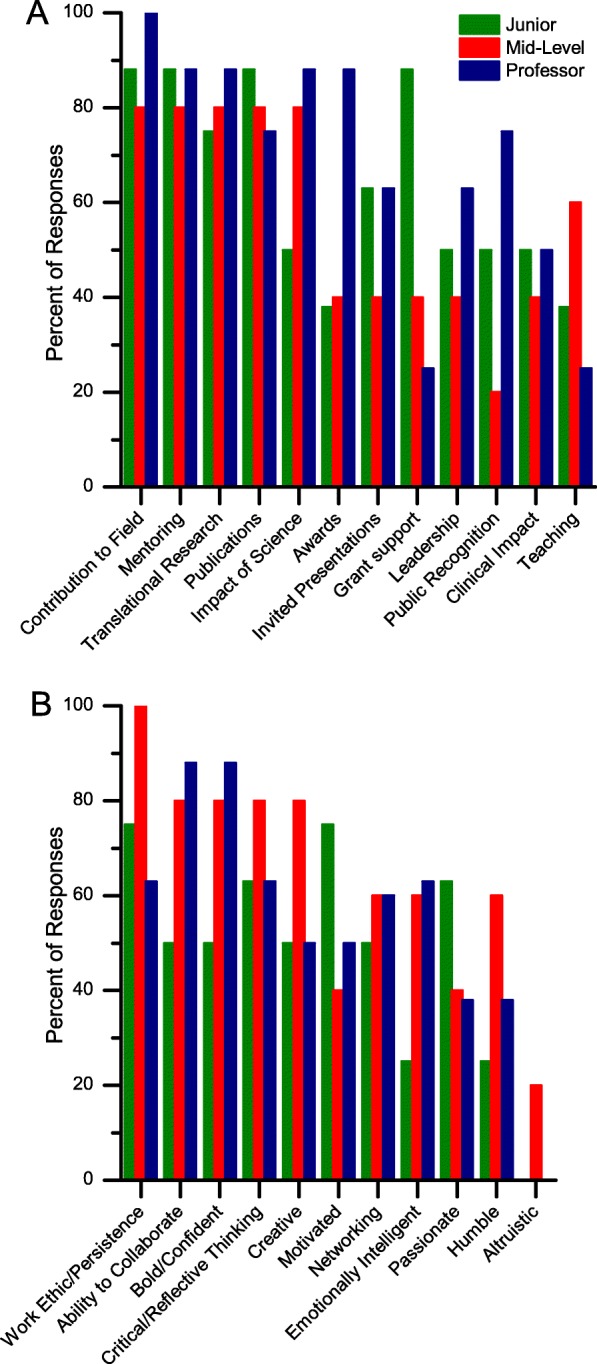
Comparison of the participants' perception of **a** objective measures and **b** subjective measures of success varied based on academic rank

More detailed examination of the responses show that the participants’ perception of success varied based on gender (Fig. 3) and academic rank (Fig. 4).

#### Breakdown by gender

Characteristics that each group found more valuable or important are described in this section as how they “ranked” the characteristics. These characteristics emanated from what they described in their interviews. Men and women had remarkably different rank orders of what constitutes success (Fig. 3). Men felt that contribution to the field was the most critical measure of success, whereas women felt it was networking.

Women ranked highly the objective and subjective characteristics of networking, contribution to the field, impact of science, awards, publications, talks at meetings, critical thinking, strong work ethic and mentoring. Men ranked highly the characteristics of contribution to the field, mentoring, engaging in translational research and publications. Among measures not listed above, women were more likely to focus on ‘Altruism’ as compared to men.

#### Breakdown by academic rank

The rank order of the measures of success also varied widely based on the participant’s rank, as summarized in Fig. 4.

Junior faculty felt that measures their promotion would be based on, such as grants and publications, were the most critical measures of success, whereas senior faculty focused more on measures related to their legacy, such as contribution to the field, mentoring, awards and invited presentations were the most important. Professors were more likely to focus on ‘Awards’ compared to other ranks. Those who had administrative responsibilities were more likely to focus on ‘Impact of publications’, ‘Grant support’, and ‘Awards’, but less likely to focus on ‘Public Recognition’.

## Discussion

Much has been written about the need to expand the physician-scientist workforce so that discoveries at the bench can be developed into cures in our lifetime [6, 7, 21, 22]. Whereas the fundamental discoveries occur at an unpredictable timeline, the translation of fundamental discoveries into clinical advances is likely to be facilitated by increasing the recruitment of trainees or by decreasing the attrition from the career path. To achieve either, it is important to have a generally agreed upon understanding of what constitutes success (in order to identify individuals who are likely to be particularly successful in this translation). To the extent that is possible, the tracking of physician-scientists (to determine whether they in fact are successful), will no longer be a moving target but a palpable goal, which in turn allows for better planning of their training.

By having a definition of success for physician-scientists, the National Institutes of Health (NIH), for example, can evaluate grants by physician-scientists using metrics that they know have a higher likelihood of predicting future success, which in turn may improve the overall training, research output and thus the nation’s health. Academic medical centers, the largest employer of physician-scientists, can focus on physician-scientist recruits who meet competencies that are recognized to increase the possibility of success and impact. Physician-scientists will benefit from this study, as they will have a clear understanding of what will be desirable in order to increase their likelihood of success.

As expected, we found no single descriptor of what constitutes success. In the overall ranking of the characteristics of success, the five predominant categories (contribution to field, mentoring, engage in translational research, quantity and quality of publications and strong work ethic/persistence) could be broken up into two categories: scholarship and work ethic. Interestingly, only two of the categories in the top five actually mention research (“contribution to the field” and “engage in translational research”). Aside from the focus on work ethic/persistence, which probably should be considered to be a predictor, rather than a descriptor of success, the top categories relate to how the physician-scientist impact others.

Within the grouping of the characteristics of a successful physician-scientist, the descriptors fell into two categories: networking/collaborating and cognitive abilities. The cognitive abilities, how we process our thinking (e.g., bold/confident, creative, critical thinking/reflecting) fall in the top third of responses whereas the communicative functions, which describe how we actually work, are rated lower.

The most common objective measures of success identified were the contribution to the field and mentoring the next generation of physician-scientists. The most common subjective measures were the physician-scientist’s work ethic, ability to collaborate and the “bold and confident manner” in which they approach their research.

The differences in responses based on gender are not surprising, even if they to our knowledge have not been described in earlier studies, because women tend to prefer relational learning [23–25]. The women were more focused on objective measures of success such as publications, while simultaneously focusing on the relational skills such as networking and collaboration — while concurrently noting the importance of public recognition. These are all qualities that could lead to increased accrual of objective measures [26–28]. Maybe reflecting the existing male-dominated culture in biomedical science, the men focused less on these objective factors and more on factors such as the impact of science and subjective characteristics such as boldness, confidence and critical thinking [29–34].

Comparing the responses for the different academic ranks revealed distinct differences in their perspectives on success of physician-scientists. Junior faculty focused on descriptors that corresponded to the criteria on which their promotion would be based upon, such as grant support and publications. Senior faculty focused on their legacy and provided a more retrospective account, which included greater emphasis on mentoring the next generation of physician-scientists. Grant support, for example, which was a top priority for the junior faculty, was a low priority for the senior faculty (but then, they would not be in the positions they were in, if they had not had ample grant support earlier in their careers). Junior faculty focused on the quantity and perceived quality of publications, whereas the senior faculty focused on the impact of the publications. This is, perhaps, the most revealing response because senior faculty seem to hire and promote junior faculty based on criteria they no longer attribute to their own success — but likely contributed to their success.

Our results must be interpreted in the context of the study design. The focus of the study was physician-scientists trained in combined MD-PhD programs in the US, and had their subsequent careers as physician-scientists in academic medical centers in the US. The elements that are included in the definition of success are likely to vary depending on the organization of the research and medical enterprises, which will vary among countries.

## Conclusion

Based on the results of this study, successful physician-scientists tend to be individuals who can be described by one or more of the following criteria:

1. Have advanced biomedical research through contributions that form the basis for future scientific breakthroughs;
2. Have made major contributions toward mentoring the next generation of (physician)-scientists;
3. Have engaged in translational research, which may have contributed directly to diagnostic or therapeutic advances;
4. Have a national reputation, as evident by their publications, invitations to speak at national and international meetings, and by being recipients of awards like the Nobel Prize, a Lasker Award, or membership in the National Academy of Sciences.

Successful physician-scientist are deemed to possess the following characteristics:

1. Maintain a strong work ethic and are persistent in pursuit of their goals;
2. Have a strong ability to collaborate on important/difficult problems;
3. Are bold and confident in their (research) decisions and thinking;
4. Think critically, and regularly reflect on their work.

Which measures of success that are considered to be most important vary based on gender and career rank. We hope this information will be useful for the MD-PhD Directors who *train* physician-scientists; the medical school deans and department chairs who *hire* physician-scientists; and NIH directors and program officers who *fund* the training of physician-scientists.

## Supplementary information

**Additional file 1: Table S1.** Summary table of objective descriptors of success based on gender and rank. **Table S2.** Summary table of subjective descriptors of success based on gender and rank.

## Data Availability

The datasets used and/or analyzed during the current study are available from the corresponding author on reasonable request. Some of the data sets are available in the supplemental data.

## Abbreviations

MSTP: Medical Scientist Training Program
NIH: National Institutes of Health
