# Supplementary material for: How perceptions of a successful physician-scientist varies with gender and academic rank: toward defining physician-scientist's success
Source: BMC Med Educ. 2020 Feb 13;20:50. doi: 10.1186/s12909-020-1960-9 (PMC7020365; doi:10.1186/s12909-020-1960-9)
Supplement: Supplementary file 1 — Additional file 1: Table S1. Summary table of objective descriptors of success based on gender and rank. Table S2. Summary table of subjective descriptors of success based on gender and rank. [file 12909_2020_1960_MOESM1_ESM.pdf]

Table S1: Summary table of objective descriptors of success based on gender and rank.

	**Gender**		**Rank**			**Total**
	Female	Male	Junior Faculty	Associate Professor	Professor	
**Objective measures**						
Contribution to the field	4 (80%)	15 (94%)	7 (88%)	4 (80%)	8 (100%)	19 (90%)
Mentoring	4 (80%)	14 (88%)	7 (88%)	4 (80%)	7 (88%)	18 (86%)
Engage in translational research	3 (60%)	14 (88%)	6 (75%)	4 (80%)	7 (88%)	17 (81%)
Quantity and quality of publications	4 (80%)	13 (81%)	7 (88%)	4 (80%)	6 (75%)	17 (81%)
Impact of science	4 (80%)	11 (69%)	4 (50%)	4 (80%)	7 (88%)	15 (71%)
Impact of publications	3 (60%)	12 (75%)	4 (50%)	3 (60%)	8 (100%)	15 (71%)
Awards	4 (80%)	8 (50%)	3 (38%)	2 (40%)	7 (88%)	12 (57%)
Invited presentations at national and int'l meetings	4 (80%)	8 (50%)	5 (63%)	2 (40%)	5 (63%)	12 (57%)
Grant support	3 (60%)	8 (50%)	7 (88%)	2 (40%)	2 (25%)	11 (52%)
Leadership/Administrative Responsibilities	3 (60%)	8 (50%)	4 (50%)	2 (40%)	5 (63%)	11 (52%)
Public recognition	3 (60%)	8 (50%)	4 (50%)	1 (20%)	6 (75%)	11 (52%)
Discoveries directly led to a clinical cure/treatment	2 (40%)	8 (50%)	4 (50%)	2 (40%)	4 (50%)	10 (48%)
Continues in same research field	1 (20%)	8 (50%)	3 (38%)	2 (40%)	4 (50%)	9 (43%)
Teaching (formal/informal)	3 (60%)	5 (30%)	3 (38%)	3 (60%)	2 (25%)	8 (38%)

Percent listed is of the category.

Table S2: Summary table of subjective descriptors of success based on gender and rank

	**Gender**		**Rank**			**Total**
	Female	Male	Junior Faculty	Associate Professor	Professor	
**Subjective measure**						
Strong work ethic/persistence	4 (80%)	12 (75%)	6 (75%)	5 (100%)	5 (63%)	16 (76%)
Ability to collaborate	3 (60%)	12 (75%)	4 (50%)	4 (80%)	7 (88%)	15 (71%)

Bold/Confident	3 (60%)	12 (75%)	4 (50%)	4 (80%)	7 (88%)	15 (71%)
Critical thinking and reflecting	4 (80%)	10 (63%)	5 (63%)	4 (80%)	5 (63%)	14 (67%)
Creative	3 (60%)	9 (56%)	4 (50%)	4 (80%)	4 (50%)	12 (57%)
Motivated	3 (60%)	9 (56%)	6 (75%)	2 (40%)	4 (50%)	12 (57%)
Networking	5 (100%)	6 (38%)	4 (50%)	3 (60%)	4 (60%)	11 (52%)
Emotionally Intelligent	2 (40%)	8 (50%)	2 (25%)	3 (60%)	5 (63%)	10 (48%)
Passionate	1 (20%)	9 (56%)	5 (63%)	2 (40%)	3 (38%)	10 (48%)
Humble	3 (60%)	5 (31%)	2 (25%)	3 (60%)	3 (38%)	8 (38%)
Altruistic	0 (0%)	1 (6%)	0 (0%)	1 (20%)	0 (0%)	1 (5%)
